# Immunoprotective Efficacy of *Acinetobacter baumannii* Outer Membrane Protein, FilF, Predicted *In silico* as a Potential Vaccine Candidate

**DOI:** 10.3389/fmicb.2016.00158

**Published:** 2016-02-12

**Authors:** Ravinder Singh, Nisha Garg, Geeta Shukla, Neena Capalash, Prince Sharma

**Affiliations:** ^1^Department of Microbiology, Panjab UniversityChandigarh, India; ^2^Department of Biotechnology, Panjab UniversityChandigarh, India

**Keywords:** *Acinetobacter baumannii*, FilF, OMP, vaccine, reverse vaccinology, cytokines, immunoprotection

## Abstract

*Acinetobacter baumannii* is emerging as a serious nosocomial pathogen with multidrug resistance that has made it difficult to cure and development of efficacious treatment against this pathogen is direly needed. This has led to investigate vaccine approach to prevent and treat *A. baumannii* infections. In this work, an outer membrane putative pilus assembly protein, FilF, was predicted as vaccine candidate by *in silico* analysis of *A. baumannii* proteome and was found to be conserved among the *A. baumannii* strains. It was cloned and expressed in *E. coli* BL21(DE3) and purified by Ni-NTA chromatography. Immunization with FilF generated high antibody titer (>64,000) and provided 50% protection against a standardized lethal dose (10^8^ CFU) of *A. baumannii* in murine pneumonia model. FilF immunization reduced the bacterial load in lungs by 2 and 4 log cycles, 12 and 24 h post infection as compared to adjuvant control; reduced the levels of pro-inflammatory cytokines TNF-α, IL-6, IL-33, IFN-γ, and IL-1β significantly and histology of lung tissue supported the data by showing considerably reduced damage and infiltration of neutrophils in lungs. These results demonstrate the *in vivo* validation of immunoprotective efficacy of a protein predicted as a vaccine candidate by *in silico* proteomic analysis and open the possibilities for exploration of a large array of uncharacterized proteins.

## Introduction

*A. baumannii* has, over the last decade, emerged as a threatening cause of bacteremia, pneumonia, septicemia, urinary tract infections, wound sepsis, endocarditis and meningitis in hospitalized patients. In certain parts of the world, it is a serious cause of community-acquired infections (Peleg et al., 2008). Although previously it was ignored as a “low-grade pathogen” due to its low virulence but its ability to cause disease and its profile of extensive drug resistance is now recognized, making *A. baumannii* an “untreatable pathogen,” especially among the patients in intensive care units (Joly-Guillou, 2005; Fournier and Richet, 2006).

*A. baumannii* is resistant to broad-spectrum cephalosporins due to overexpression of the chromosomal AmpC-type cephalosporinase (Corvec et al., 2003; Rodriguez-Martinez et al., 2010). Additionally, there are frequent reports of acquired resistance (Coelho et al., 2004) to all beta-lactams, mainly due to enzymatic degradation by carbapenem hydrolyzing beta-lactamases. Resistance to fluoroquinolones and aminoglycosides is also very common (Coelho et al., 2004; Peleg et al., 2008), facilitating its adaptation to environmental selection pressure and leading to the rapid worldwide emergence of multidrug-resistance. As a last resort, there has been increased use of antibiotics such as colistin (Li et al., 2006; Peleg et al., 2008), unfortunately leading to the emergence of colistin-resistant strains (Adams et al., 2009; Rolain et al., 2011; Qureshi et al., 2015). The extensive drug resistance of this pathogen and the predictable failure of future antibiotic treatment options warrant the development of vaccine against *A. baumannii*.

Several attempts have provided immunological insights to such treatment options against *A. baumannii* infections e.g., monoclonal antibodies against the iron regulated outer membrane proteins (IROMPs) were found bactericidal and exhibited opsonizing activities during *in vitro* studies (Goel and Kapil, 2001). Active and passive immunization with inactivated whole cell (McConnell and Pachón, 2010), outer membrane vesicles (OMVs) (McConnell et al., 2011; Huang et al., 2014) and outer membrane complexes (OMCs) (McConnell et al., 2011) demonstrated protection of mice from bacterial challenges. Sub-unit vaccine candidates such as Bap (Fattahian et al., 2011), rOmpA (Luo et al., 2012), Ata (Bentancor et al., 2011) and nuclease (Garg et al., 2016) have been found to provide protection against pathogenic strains. Recently, Moriel et al. (2013) and Chiang et al. (2015) reported a few vaccine candidate proteins in the outer membrane and secretome, and immunization with OmpK, FK IB and Ompp1 provided partial protection from *A. baumannii* ATCC 17978. In spite of all these studies, there is no vaccine-based treatment available to prevent *A. baumannii* infections.

There are numerous proteins that need to be explored for their role in virulence and pathogenesis of *A. baumannii* and also for their vaccine potential. In this work, *in silico* analysis of *A. baumannii* ATCC 19606 proteome predicted FilF, an outer membrane, uncharacterized putative pilus assembly protein, as a potential vaccine candidate. It was found to be conserved though its role in virulence is not yet known. FilF was cloned, purified and analyzed by *in vitro* and *in vivo* experiments in a murine pneumonia model for its immunoprotective efficacy.

## Materials and methods

### Animals, ethical clearance, and bacterial strains

Pathogen-free, 6–8 weeks old female Balb/c mice were procured from animal house, Panjab University, Chandigarh, India and housed in clean polypropylene cages and fed a standard antibiotic-free diet (Hindustan Lever Products, Kolkata, India) and water *ad libitum*. Animal studies were approved by the Animal Ethics Committee of Panjab University, Chandigarh, India. All experiments were performed in accordance with the guidelines of Committee for the Purpose of Control and Supervision of Experiments on Animals (CPCSEA), Government of India. All efforts were made to minimize the suffering of animals.

*A. baumannii* ATCC 19606 was procured from ATCC and was used to establish murine pneumonia model. *E.coli* BL21 (DE3) and pET28-a plasmid from Novagen were used for cloning and expression of FilF. The bacterial strains were grown in Luria-broth (LB) containing kanamycin (25 μg/ml), wherever required.

### *In silico* analysis of *A. baumannii* ATCC 19606 proteome

Complete proteome of *A. baumannii* ATCC 19606 was downloaded from NCBI nucleotide database and analyzed for potential vaccine candidates using the Vaxign online tool (He et al., 2010) by searching (i) localization in outer membrane using PSORTb, (ii) number of trans-membrane helices using HMMTOP, (iii) adhesion probability using SPAAN, (iv) no similarity with human and mouse proteome using OrthoMCL, and (v) ability to bind to MHC molecules using Vaxitop. B cell, MHC I and MHC II binding epitopes were predicted by IEDB tools (www.iedb.org). ProtParam was used to analyze the physico-chemical parameters such as molecular weight, hydropathicity, pI and stability. Phyre2 and GOR IV online tools were used to predict the secondary structure of FilF. Tertiary structure of FilF was generated using I-TASSER online tool based on homology modeling. Procheck (Ramachandran Plot) and verify 3-D were used to validate the quality of generated structure.

### Cloning and purification of FilF

Chromosomal DNA of *A. baumannii* ATCC 19606 was isolated (Sambrook and Russell, 2001) and used as template for PCR. Primers were designed by online tool “OligoEvaluator™” for *filF* (Accession ID- EEX03804.1, the 6th ORF in *fil* operon, Supplementary Table S3) having BamHI and XhoI restriction sites in forward (5′- ATA**GGATCC**TGTGGTGGAGGAAGTT-3′) and reverse primer (5′- TCA**CTCGAG**TTATTTTGTCTTAATTTGATAACAAT-3), respectively. PCR reaction was performed with initial denaturation at 94°C for 3 min followed by 33 thermal cycles of denaturation at 95°C for 1 min, annealing at 55°C for 45 s, and extension at 72°C for 2 min. Final extension was carried out at 72°C for 5 min. The BamHI and XhoI digested PCR product was ligated to similarly digested pET-28a and transformed into *E. coli* BL21 (DE3) by electroporation. Transformants were selected on LB-kanamycin agar plates and confirmed by PCR.

Five hundred milliliter of LB-kanamycin was inoculated with 2.5 ml of overnight grown culture of *E. coli* BL21 (DE3) containing pET-28a-*filF*. Isopropyl β-D-1-thiogalactoside (IPTG) (0.5 mM) was added when OD_600_ reached 0.8 and induced for 5 h at 37°C/150 rpm. The cells were pelleted and suspended in 50 ml lysis buffer (100 mM phosphate buffer, 300 mM NaCl, 0.2% Tween 20, pH 8) containing 1 mg/ml lysozyme. The cell suspension was sonicated, centrifuged and pellet was solubilized in 100 mM phosphate buffer, pH 8 containing 8 M urea and 300 mM NaCl. The lysate was centrifuged at 13,000 × g for 40 min and supernatant was filtered (0.45 μm) and loaded on Ni-NTA column equilibrated with equilibration buffer (8 M urea, 20 mM Tris–HCl, 100 mM phosphate buffer, pH 8.0). The nonspecific proteins were removed by washing with five column volumes of wash buffer (8 M urea, 20 mM Tris–HCl, 500 mM NaCl, pH 6.9). The bound FilF was eluted with buffer containing 8 M urea, 20 mM Tris–HCl, 500 mM NaCl, 100 mM phosphate buffer, pH 4.5. Eluted fractions were collected and analyzed by 12% SDS PAGE (Laemmli, 2011). The protein was refolded by urea gradient dialysis method according to Qiagen's guidelines. Protein concentration was estimated by Bradford protein estimation kit (Bangalore Genei India Pvt. Ltd.). The endotoxin level of purified recombinant FilF was determined using Limulus Amebocyte Lysate assay kit (Hycult Biotech, The Netherlands) according to manufacturer's guidelines.

### *A. baumannii* associated pneumonia model

*A. baumannii* associated mouse pneumonia model was established by intratracheal route. Briefly, *A. baumannii* ATCC 19606 was grown in LB broth to late-logarithmic phase at 37°C/150 rpm. Cells were harvested by centrifugation at 6000 × g for 10 min, washed and resuspended in PBS. Different doses of bacteria (10^6^–10^9^ CFU) were obtained by appropriate dilutions and the final cell count was quantified by plating serial dilutions on LB agar plates. Mice were anesthetized with a mixture of xylazine, ketamine and PBS in the ratio 6:1:3, respectively by injecting intraperitoneally. Desired dose of bacteria in a total volume of 50 μl was inoculated directly in trachea by surgery. The incisions were sealed using surgical sutures and betadine was applied on cuts to prevent infections. Group of mice inoculated with PBS served as control. At specific time intervals mice were sacrificed, lungs were isolated aseptically and homogenized for histology and to determine the bacterial counts.

### Mouse immunization and antibody titer measurement by ELISA

FilF specific antibodies were produced in mice according to McConnell et al. (2006). Briefly, the concentration of refolded protein was adjusted to 0.4 mg/ml in sterile PBS and diluted 1:1 (v/v) with Freund's Complete Adjuvant (Sigma). 100 μl of protein-adjuvant mixture was administered in mice sub-cutaneously (20 μg FilF per mouse). Booster doses of protein were given with Freund's Incomplete Adjuvant (Sigma) at 14th and 21st day and sera were collected at day 7, 18, and 25. FilF specific IgG antibodies were measured by ELISA. Briefly, 200 ng FilF in sodium bicarbonate buffer (pH 9.6) was coated to each well by incubation at 4°C overnight. The wells were washed thrice with 0.1% Tween 20 in PBS (PBST) and blocked with 5% BSA in PBST (PBSTM) for 1 h at room temperature. Sera were serially diluted two fold in PBSTM and added to wells followed by incubation for 1 h at 37°C. Wells were washed thrice with PBST and 100 μl of horseradish peroxidase-conjugated anti-IgG (Bangalore Genei) diluted in PBSM (1:5000) was added to each well and incubated at room temperature for 1 h. Wells were again washed with PBST thrice and 100 μl of horseradish peroxidase substrate (Bangalore Genei India Pvt. Ltd.) was added to each well and developed for 20 min at room temperature. The reaction was stopped with the addition of 100 μl of 2M HCl, and the absorbance was read at 450 nm on an ELISA reader (BioRad). The endpoint titer was defined as the highest dilution at which the optical density at 450 nm was significantly higher than that of control wells receiving control adjuvant serum.

### Serum cytokine's levels estimation

Sera were collected from adjuvant control and FilF immunized mice at 12 and 24 h post infection. Serum levels of cytokines TNF-α, IL-6, IL-33, IFN-γ, IL-1β, and IL-10 were estimated using Krishgen Biosystems, India and GenAsia, Philippines kits according to the supplier's instructions. Concentrations were calculated in Graphpad Prism 5 software.

### Bacterial load in lungs

Mice were sacrificed by cervical dislocation and dissected aseptically to remove lungs. The lungs were suspended in 1ml PBS and homogenized. Homogenates were serially diluted and spread plated on Luria agar followed by incubation at 37°C overnight. The number of colony forming units was counted and the results were expressed as log CFU.

### Histopathology examination

Aseptically collected lung specimens were fixed in 10% buffered formalin, stained with hematoxylin-eosin and observed under microscope at 100X magnification.

### Statistical analyses

All statistical analyses were performed using Graphpad Prism 5 software. The data were presented as mean with standard deviations represented as error bars. One way analysis of variance (ANOVA) was applied for all the comparisons. Survival rates were analyzed by log-rank test. Results were considered significant at *p* < 0.05.

## Results

### Prediction of FilF as vaccine candidate using vaxign

Complete proteome analysis of *A. baumannii* yielded 57 proteins, predicted as vaccine candidates by Vaxign. An uncharacterized protein FilF was selected for further analysis as it was conserved among the strains of *A. baumannii*. Physical and chemical parameters of FilF were computed using ProtParam online tool (Supplementary Table S1). FilF is 641 amino acid protein having signal peptide of 20 amino acids which directs its localization to outer membrane. Its predicted molecular weight is ~68kDa with pI 5.21. It possesses high adhesion probability (*p* = 0.879), no trans-membrane helix and no similarity to human and mouse proteome. Protein-BLAST of FilF showed that it matched and belonged exclusively to *Acinetobacter* and was present in the sequenced genomes (complete and drafted) of 250 strains of *Acinetobacter* available in UniProt database. It shared >99% similarity with 25 strains of *A. baumannii* (Supplementary Table S2), further FilF shared 50–90% in other strains and 35–50% identity in other species of *Acinetobacter*. Though similarity levels vary but majority of epitopes (B cell, MHC I and MHC II) fall in these identical regions. Upstream and downstream DNA sequences showed that *filF* is regulated in *fil* operon and there are 5 other *fil* genes having different functions (Supplementary Table S3). Secondary structure prediction (Supplementary Figure S1) revealed that FilF is composed of mainly random coils (369 amino acids), extended strands (153 amino acids) and alpha helices (119 amino acids). Homology modeling was done using I-TASSER and 3-D structure was predicted (Supplementary Figure S2). FilF shared no structural similarity in Protein Data Bank (PDB) and I-TASSER used pilus adhesin (RrgA) from *Streptococcus pneumoniae* as the closest template to generate a 3D structure for FilF but quality of predicted structure was not acceptable as 13 residues out of 641 amino acids of FilF fell into disallowed region of Ramachandran plot (Supplementary Figure S3). B cell, MHC I and MHC II binding epitopes for the HLA alleles prevalent in North India were predicted by IEDB (Supplementary Tables S4–S6, respectively) which supported the strong FilF immunogenicity.

### FilF formed inclusion bodies

**Figure 1:**
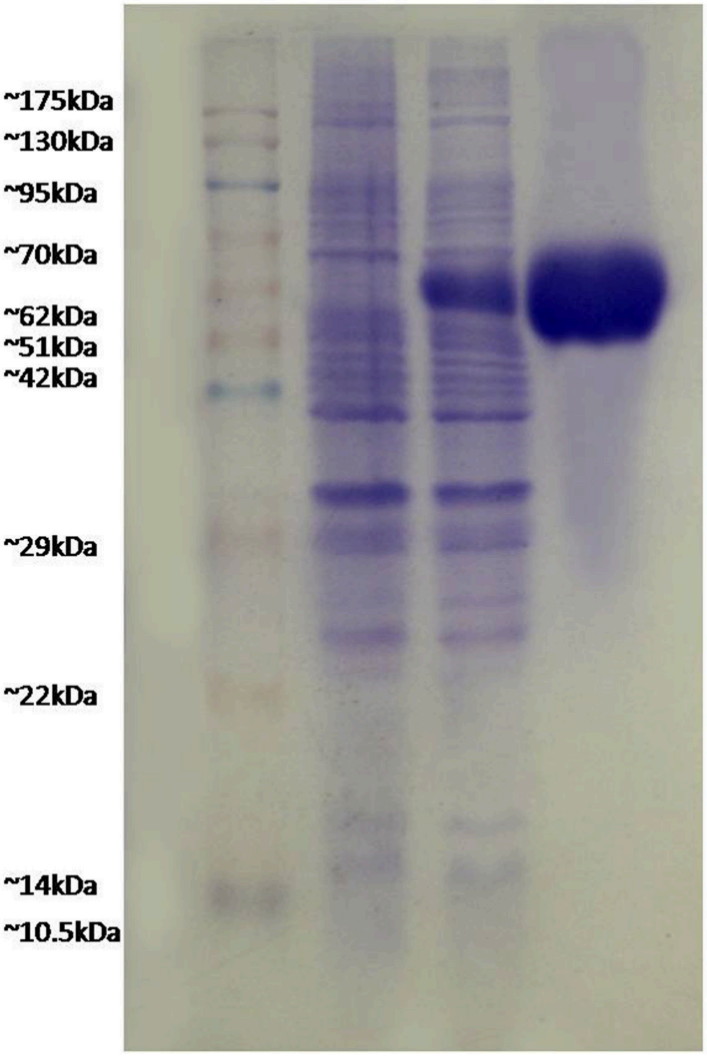
**Expression and purification of FilF protein**. Lane 1, pink plus marker; lane 2, uninduced sample; lane 3, induced FilF; lane 4, purified FilF protein.

FilF was cloned in pET-28a (Supplementary Figure S4) and expressed in *E.coli* BL21 (DE3). Due to over expression, FilF formed inclusion bodies and accumulated in insoluble fraction. Prevention of inclusion bodies was tried by optimizing IPTG concentration (0.01to 1 mM), temperature (15°C to 37°C) and time (1–16 h) but protein still accumulated as inclusion bodies which were dissolved in 8M urea and purified by Ni-NTA chromatography up to a concentration of 40 mg/L. Purified protein was refolded by urea gradient dialysis and resolved on SDS-PAGE (Figure 1). The endotoxin level of purified recombinant protein used for immunization was found to be <1 EU/ml.

### FilF-specific antibodies

**Figure 2:**
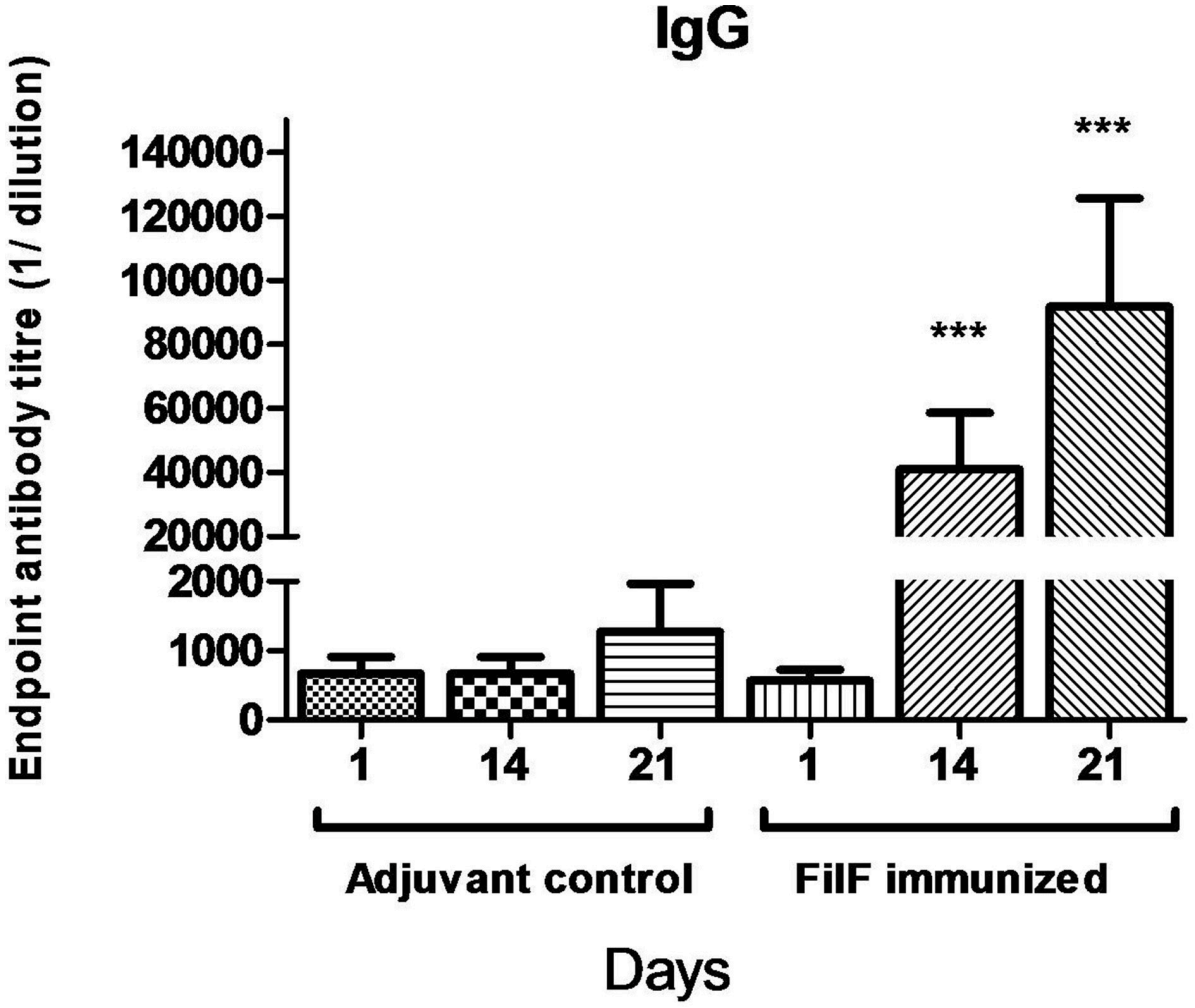
**Total IgG antibody levels in the sera**. Groups of female BALB/c mice (*n* = 10) were sub-cutaneously immunized with 20 μg FilF formulated with CFA/IFA at day 1, 14, and 21. Sera from adjuvant control and FilF immunized mice were collected 3 days after booster dose and IgG titer was determined. Antibody response at 14 and 21-day rose as compared to adjuvant control. ^***^p < 0.001 (Adjuvant control vs. FilF immunized mice).

In order to evaluate the antibody response to immunization with the FilF protein, mice (*n* = 10) were immunized at day 1, 14, and 21 with 20 μg of purified FilF protein. Significant levels of IgG antibody titer were observed after each booster in the sera of immunized mice as compared to adjuvant control mice (Figure 2). Antibody titer significantly increased to >6.4 × 10^4^ after second booster. The adjuvant control mice did not show any FilF-specific IgG response at any time point.

### Effect of FilF immunization on bacterial load in lungs

Different bacterial doses (10^6^–10^9^ CFU) of *A. baumannii* ATCC 19606 were intratracheally administered to groups of mice and effects were observed. Mice showed mild clinical symptoms postinfection with 10^6^ and 10^7^ CFU of bacteria which were cleared from the body within 2–3 days (data not shown). 10^8^ CFU caused infection resulting in mice death within 24–48 h and this dose was selected for further experiments.

**Figure 3:**
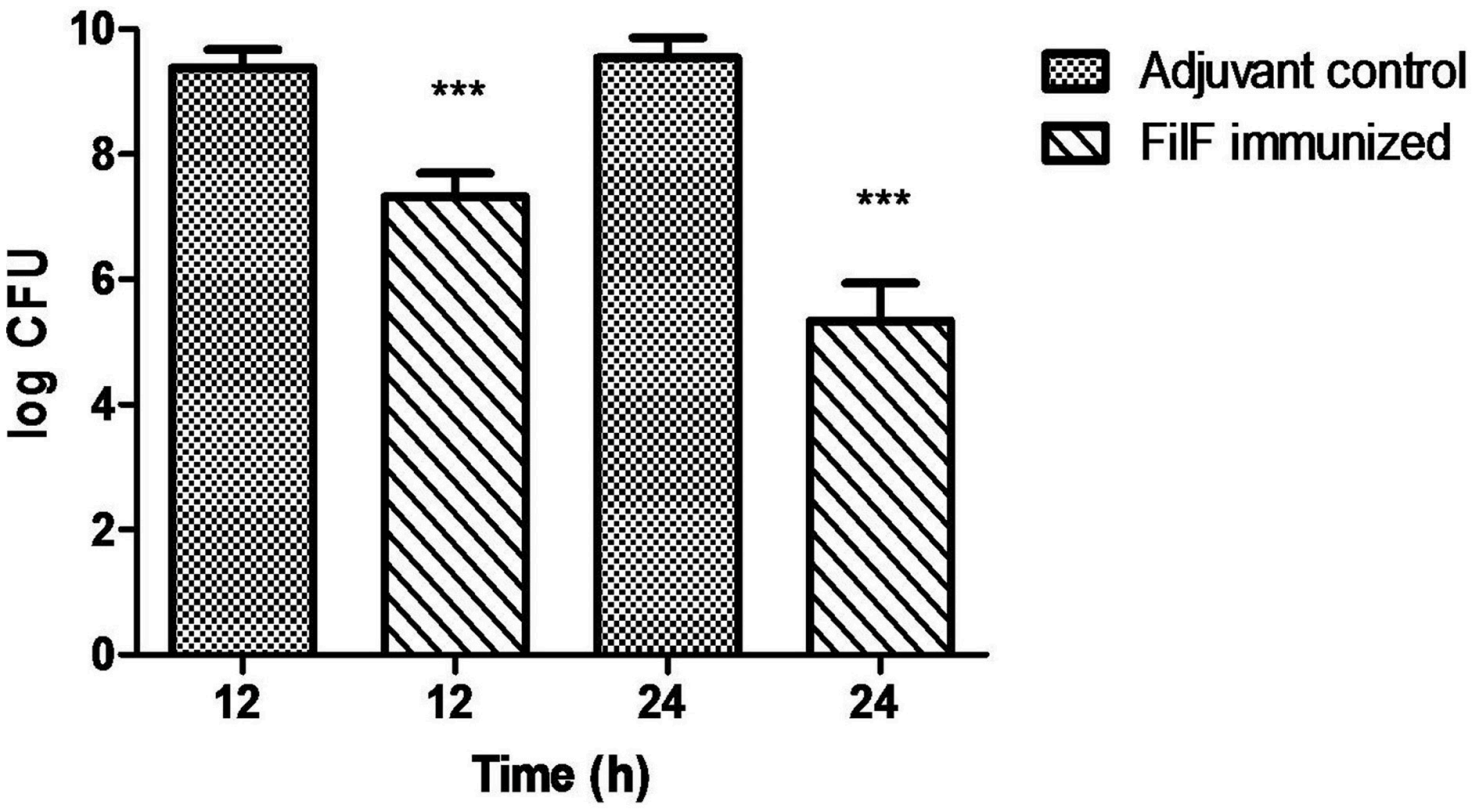
**Bacterial burden in lungs**. Groups of female Balb/c mice (*n* = 6) were immunized subcutaneously with 20 μg FilF formulated with CFA/IFA adjuvant on day 1, 14, and 21. The mice were intra-tracheally challenged with 10^8^ CFU of *A. baumannii* ATCC 19606 at day 29. Immunization with FilF reduced the bacterial burden by 2 and 4 log cycles in the lungs of pneumonia model mice sacrificed 12 and 24 h post infection, respectively. The data are presented as mean ± SD (*n* = 6). *p*-value was determined by the one way analysis of variance (ANOVA). ^***^p < 0.001 (Adjuvant control vs. FilF immunized mice).

Using the developed murine pneumonia model, the effect of FilF immunization on bacterial load was determined by quantifying bacteria in the lungs of unimmunized and FilF immunized mice 12 and 24 h after infection with 10^8^ CFU of the *A. baumannii* ATCC 19606. Bacterial load was lowered by only 2 log cycles 12 h postinfection but it showed significant reduction by 4 log cycles 24 h postinfection as compared to adjuvant control mice (Figure 3).

### Histological examination

**Figure 4:**
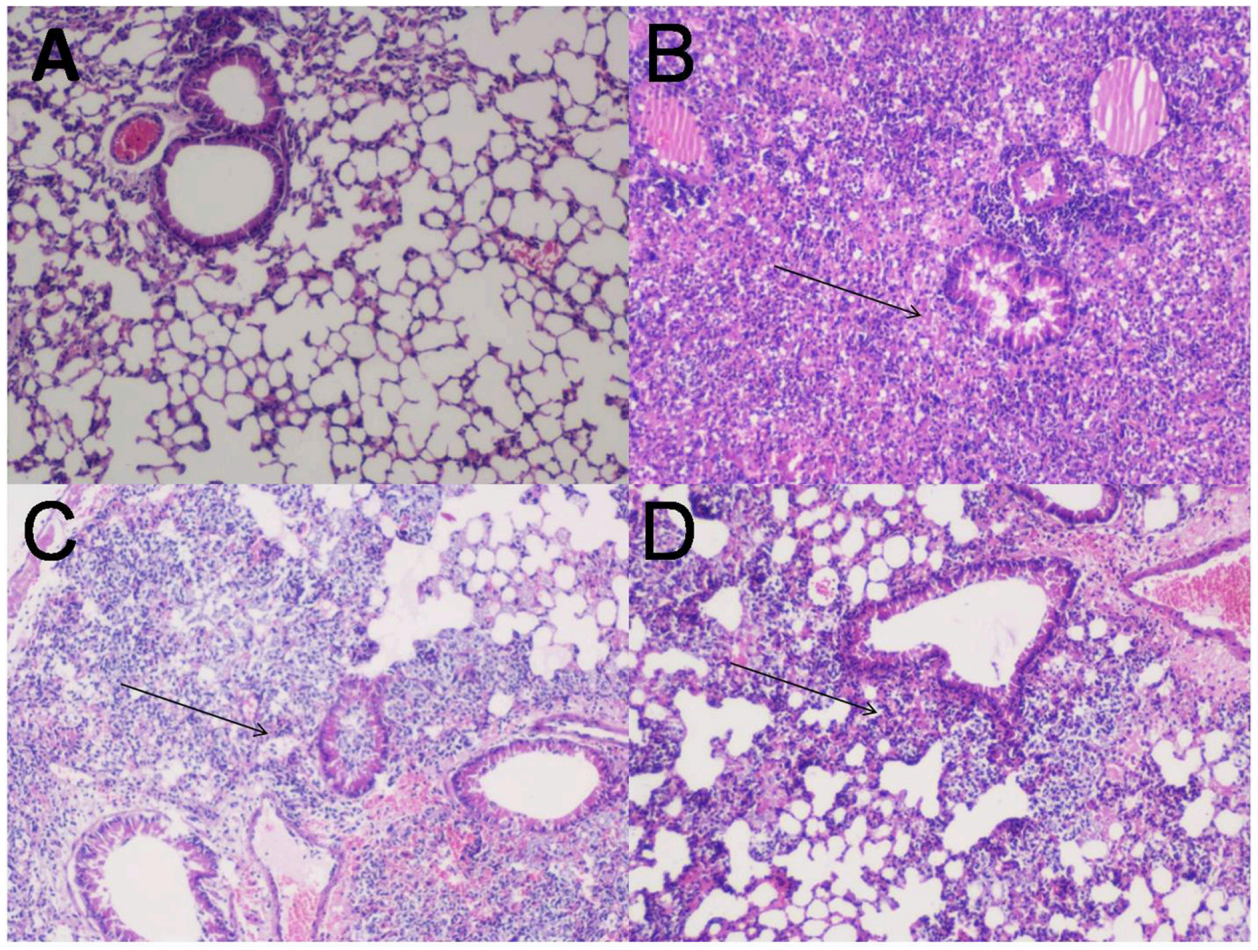
**Lung histopathology**. Groups of female Balb/c mice (*n* = 6) were immunized subcutaneously with 20 μg FilF formulated with CFA/IFA adjuvant on day 1, 14, and 21, and intra-tracheally challenged with 10^8^ CFU of *A. baumannii* ATCC 19606 at day 29. The mice were sacrificed at 12 and 24 h post-challenge and lungs were collected for histopathology. **(A)** The lung from an unimmunized uninfected mouse showing normal histological characters. **(B)** Unimmunized infected mouse lung showing increased inflammatory cell infiltration in the perivascular and peribronchial areas, and within the airway lumen (arrows) 12 h postinfection. **(C)** The lung from an immunized infected mouse showing mild inflammatory cell infiltration in the perivascular and peribronchial areas (arrows) 12 h postinfection. **(D)** The lung from an immunized infected mouse showing significantly reduced infiltration of inflammatory cells 24 h postinfection. H&E, Magnification 100X.

Lungs of normal uninfected, adjuvant control and FilF immunized mice were removed 12 and 24 h post-infection, stained and visualized for histopathological changes (Figure 4). Bacterial challenge caused pneumonia and bacterial consolidation in the unimmunized mice. Lungs were filled with the increased number of lymphocytes and neutrophils 12 h post infection in unimmunized mice whereas immunized mice had moderate inflammation with infiltration of mixed mononuclear cells and neutrophils around peribronchial and perivascular areas. Moreover, lungs of immunized mice appeared normal with small number of neutrophils 24 h postinfection indicating that FilF immunization was able to limit the infection.

### Effect of immunization on serum cytokines levels

**Figure 5:**
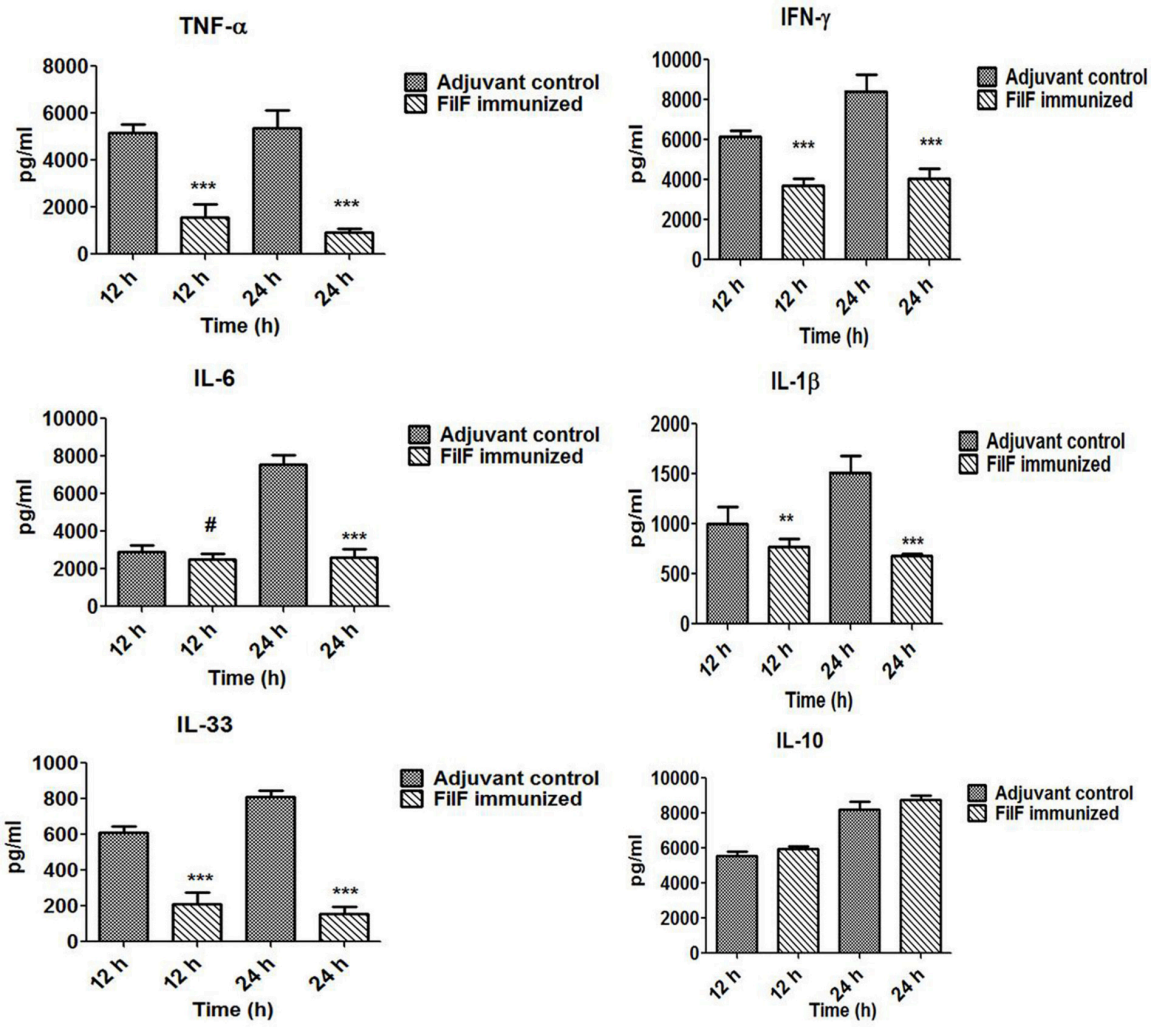
**Cytokine levels in the sera**. Groups of female Balb/c mice (*n* = 6) were immunized subcutaneously with 20 μg FilF formulated with CFA/IFA adjuvant on day 1, 14, and 21. The mice were intra-tracheally challenged with 10^8^ CFU *A. baumannii* ATCC 19606 at day 29, and sacrificed at 12 and 24 h post-challenge. The detection limit for all cytokines and chemokines is <10 pg/ml. *p*-value was determined by the one way analysis of variance (ANOVA). ^*^p < 0.1, ^**^p < 0.05, ^***^p < 0.001, ^#^non-significant, (Adjuvant control vs. FilF immunized mice).

Levels of pro- and anti-inflammatory cytokines were determined to check whether FilF immunization was able to prevent the release of these cytokines (Figure 5). Serum levels of pro-inflammatory cytokines TNF-α (*p* < 0.001), IFN-γ (*p* < 0.001) and IL-1β (*p* < 0.05) were found to be significantly lower in immunized mice 12 h postinfection whereas IL-6 and anti-inflammatory cytokine IL-10 levels were comparable with unimmunized adjuvant control mice group. After 24 h postinfection, levels of all the pro-inflammatory cytokines TNF-α (*p* < 0.001), IFN-γ (*p* < 0.001) and IL-1β (*p* < 0.001) were significantly low in immunized mice. However, IL-6 levels increased in adjuvant control and remained low in FilF immunized mice group 24 h postinfection (*p* < 0.001) (Figure 5). IL-10 levels remained comparable to unimmunized mice and the change was non-significant 24 h postinfection. Levels of IL-33, associated with the inflammatory responses of lung tissues, were also determined and interestingly, FilF immunized mice showed significantly low levels (*p* < 0.001) as compared to unimmunized mice 12 and 24 h postinfection.

### Survival against challenge with lethal dose of *A. baumannii*

**Figure 6:**
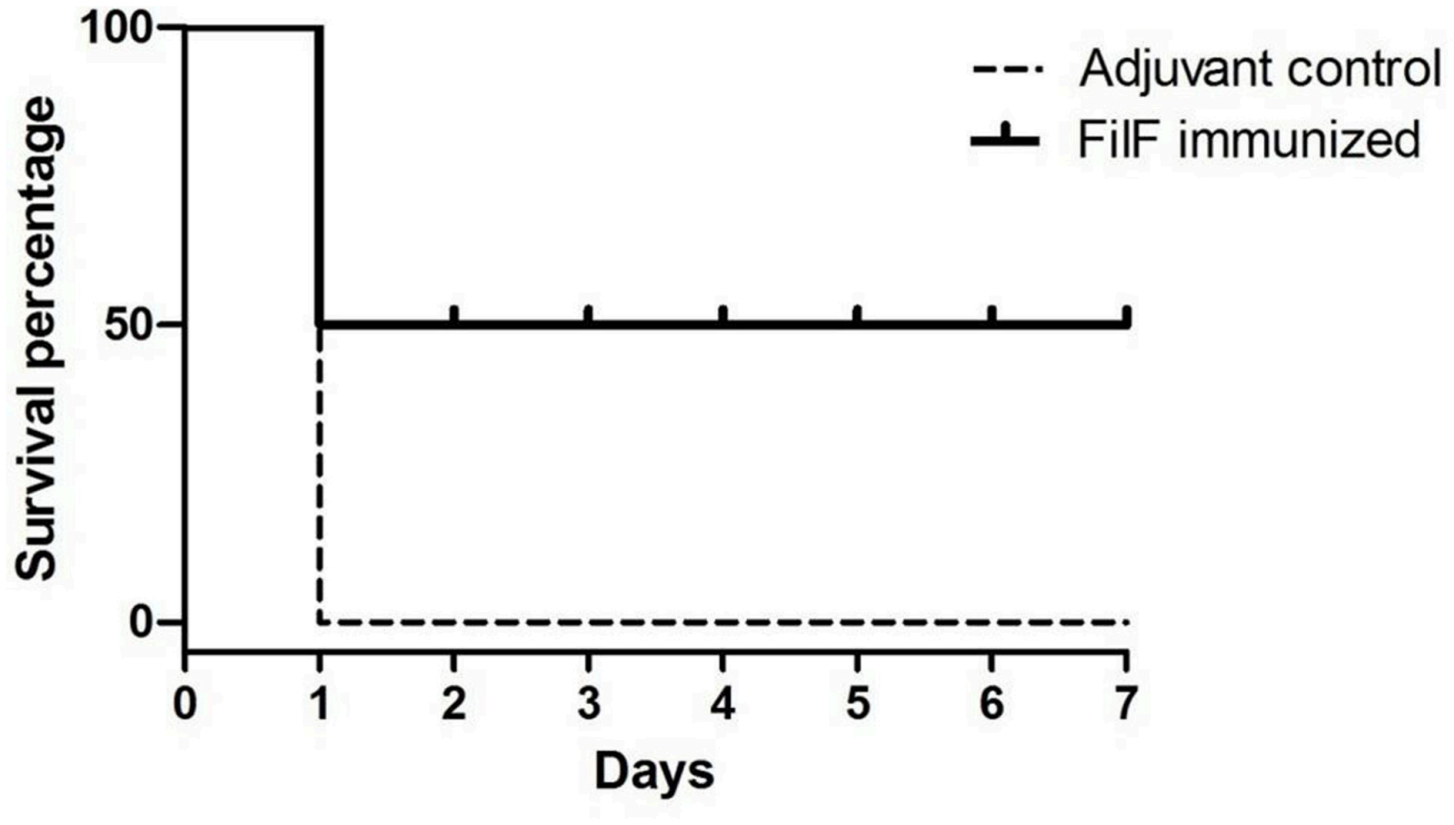
**Survival rate of mice**. Groups of female Balb/c mice (*n* = 10) were immunized sub-cutaneously with 20 μg FilF formulated with CFA/IFA adjuvant on day 1, 14, and 21, and intra-tracheally challenged with 10^8^ CFU of *A. baumannii* ATCC 19606 at day 29. The survival rates of mice were recorded for seven days.

Effectiveness of FilF immunization was determined by determining the survival rate after challenge with lethal dose of *A. baumannii*. Groups of mice (*n* = 10) were immunized sub-cutaneously with 20 μg FilF formulated with CFA/IFA adjuvant on day 1, 14, and 21, and intra-tracheally challenged with 10^8^ CFU of *A. baumannii* ATCC 19606 at day 29. The survival rate of mice was recorded continuously over the next seven days. FilF immunized (*n* = 10) mice showed improved survival rate as compared to unimmunized mice (*n* = 10) after challenge. All adjuvant control mice died within 24–48 h whereas FilF immunized mice showed 50% survival rate observed till seven days (Figure 6).

## Discussion

Advances in bioinformatics have opened new possibilities for rapid identification of vaccine candidate proteins against MDR pathogens. Reverse Vaccinology (Rappuoli, 2001) has emerged as a robust method for identifying subunit vaccines and has been successfully used to develop vaccines against pathogens viz. *Neisseria meningitidis* (Pizza et al., 2000), *Porphyromonas gingivalis* (Ross et al., 2001), *Streptococcus pneumoniae* (Maione et al., 2005), Hepatitis C virus (Sarbah and Younossi, 2000) and *Mycobacterium tuberculosis* (Ridzon and Hannan, 1999) showing its potential to be tested on the other emerging pathogens such as *A. baumannii* which is gaining attention of the medical world due to the rapid acquisition of multidrug resistance. Development of nonspecific immune response and chances of reverting back to virulent form dissuade the use of inactivated whole cells or outer membrane complexes. To generate specific immune response, outer membrane proteins such as porins, lipoproteins, ton-b receptors, biofilm associated proteins, transporter proteins or pilus proteins can be assessed as single subunit vaccine candidates. These outer membrane proteins are recognized as foreign by host immune system and are potential vaccine candidates against pathogens.

FilF is such a candidate protein predicted by *in silico* analysis of *A. baumannii* proteome. It is a putative pilus assembly protein and exact role in virulence is unknown but presence of FilF in virulent clinical isolates and in the outer membrane vesicles of invasive clinical strains makes it a crucial antigen (Mendez et al., 2012; Li et al., 2015). Bioinformatic analysis shows its localization in outer membrane, no transmembrane helices, high adhesion probability, conservation among the different strains of *A. baumannii*, presence of epitopes specific to the HLA alleles prevalent in north India (Rani et al., 2007) and dissimilarity with human and mouse proteome, thus presenting it as a highly potential vaccine candidate. B cell and T cell (MHC–I and MHC–II) epitopes in FilF were determined in order to assess its antigenicity and to look for the epitopes prevalent in north Indian population. Though FilF is well conserved among *A. baumannii* strains, yet determining the absolutely conserved regions containing the highly antigenic epitopes will tell about the protective immunity against all strains of *A. baumannii* and may lead to generation of a peptide vaccine by epitope stitching, which could be used to elicit immunity against a group of gram negative co-infecting pathogens.

To monitor immunoprotecive efficacy of FilF, a murine pneumonia model was established via intratracheal route. In case of *A. baumannii*, respiratory tract is the most common site of infection and colonization. Administration of bacteria to lungs through intranasal route produces bronchopneumonia and intratracheal route results in lobar pneumonia resulting in rise in cytokine levels and mice death (Joly-Guillou et al., 1997). But a major limitation of *A. baumannii* infection models (pneumonia and sepsis) is that mice clear the large proportion of the pathogen. Intranasal route was tried to establish the infection in mice with moderate (10^5^) to high (10^8^) CFU count but bacteria were cleared without causing the infection (results not shown). Later, intratracheal administration of optimized lethal dose of bacteria i.e., 10^8^ CFU into mice caused infection resulting in mice death within 24–48 h. Such high dose (10^8^ CFU) of *A. baumannii* could match the pathogenesis and virulence level of even the most virulent strain. Challenge with 10^8^ CFU caused severe pulmonary infection in unimmunized mice and caused 100% mortality. FilF immunization elicited high humoral response resulting in decreased bacterial load, reduced levels of pro-inflammatory cytokines and increased survival rate (50%) by controlling the severity of infection. Although survival rate of more than 50% has been reported by immunization with crude cell extract or outer membrane vesicles (McConnell et al., 2011; Huang et al., 2014) but the immunoprotective efficacy of subunit recombinant FilF is significant. Besides, bacterial burden in lungs was also significantly reduced by 2 and 4 log cycles, 12 and 24 h post challenge, respectively. This considerable reduction in bacterial loads is indicator of FilF efficacy. OMVs have FilF as a component and are effective vaccine formulations, but because of the complications of solubility, variability and low prevalence of antigens in OMVs, exploration of other vaccine candidates which may provide broad spectrum protection against this evolving pathogen is required. Identification and evaluation of individual vaccine candidates through immunoinformatics analysis of proteomes is an ideal choice for rational vaccine development. Recombinant FilF as antigen has advantages over inactivated whole cell or outer membrane vesicles such as the high levels of antigen purity, easy and reproducible large scale protein production, generation of specific immune response and absence of contaminating bacterial components such as lipopolysaccharide that could produce unwanted side effects. These attributes assist in the approval from regulatory agencies. OMVs contain many proteins which could affect the immune response generated by the effective antigens present in the formulation.

These results were further supported by histological evaluation of lungs. *A. baumannii* associated pneumonia can be identified by various disorders in the lung such as inflammation, bronchitis, abscess and edema. Oxidative stress and inflammation induced by this pathogen contribute to the death of lung epithelial cells (Smani et al., 2011). Reduced neutrophil infiltration in immunized mice as compared to unimmunized controls showed the efficacy of FilF. Interestingly, levels of IL-33 were significantly lower in immunized mice. IL-33, a critical mediator of the innate immune response, is released by epithelial cells on invasion by pathogen and initiates the immune response. It is supposed to play role in the recruitment of immune cells to the lungs and promotes bacterial clearance from the lungs of mouse in pneumonia model (Huang et al., 2014). Also, during infection, inflammation of lungs followed by increase in pro-inflammatory cytokine levels occurs due to the bacterial virulence factors such as pilus, porins, outer membrane proteins, lipopolysaccharides, capsular polysaccharides, proteases and nucleases. Pro-inflammatory cytokines such as TNF-α, IL-1β, and IL-6 have been reported to play important role in cell death induced by *A. baumannii*. TNF-α binds to its specific receptor and initiates the caspase 8, 10, and 3 mediated apoptosis whereas IL-6 modulates expression of pro- or anti-apoptopic factors involved in the activation of intrinsic pathways of apoptosis (Smani et al., 2011). IFN-γ is a critical cytokine for innate and adaptive immunity against viruses and some bacterial pathogens. It activates macrophages and induces MHC II molecules (Schoenborn and Wilson, 2007). In this study, significant increase in the levels of pro-inflammatory type 1 (TNF-α, IFN-γ, IL-6, IL-1β), pro-inflammatory type 2 (IL-33) and anti-inflammatory type 2 cytokines was observed in unimmunized mice after bacterial challenge indicating the spread of infection. Increase in levels of pro-inflammatory cytokines has been observed in other immunoprotective studies and is related to the high bacterial load in organs (McConnell et al., 2010, 2011; Huang et al., 2014). The strong inflammatory response in unimmunized mice was possibly due to high bacterial challenging dose. FilF immunization, but not the adjuvant control, was able to reduce the levels of pro-inflammatory cytokines significantly (Figure 4), that controlled the infection and decreased the damage to lung cells. But the levels of anti-inflammatory cytokine IL-10 remained comparable to unimmunized controls even after 24 h postinfection.

Antibody response is also a critical indicator to assess the effectiveness of a vaccine and FilF immunization evoked a high humoral response in mice. IgG antibody titer after first booster was >32,000 and after second booster reached >64,000. Anti-FilF antibodies may bind to *A. baumannii* surface and promote opsonization, phagocytosis and killing of the pathogen by macrophages and neutrophils. Moreover, antibody-mediated complement activation can lead to lysis of the pathogen and can also induce localized production of immune effector molecules that help to develop an amplified and more effective inflammatory response. FilF is a part of the specialized secretion system for the delivery of virulence factors in *A. baumannii* (Mendez et al., 2012). Anti-FilF antibodies could have bound to FilF and prevented the delivery of bacterial virulence factors to the host. Also, FilF is a putative pilus assembly protein, so anti-FilF antibodies, in addition to T cell activation, may bind to FilF and prevent the attachment of *A. baumannii* to the lungs of mouse, an initial and crucial step in establishment of *A. baumannii*, thus resulting in the enhanced survival of mice after challenge.

This is the first report on FilF immunoprotective efficacy and supports the significance of *in silico* prediction and *in vivo* validation of FilF, elicting strong protective response against *A. baumannii*. Compared to the earlier vaccine development efforts, FilF can be considered a promising vaccine candidate against infections caused by *A. baumannii*.

## Conclusion

This work shows that FilF (i) is conserved among the strains of *A. baumannii* (ii) is a potential vaccine candidate predicted by *in silico* analysis (iii) raises high antibody titer in mouse (>64,000), (iv) reduces cytokine response [significant reduction in pro-inflammatory cytokines TNF-α, IL-33, IFN-γ, IL-6, and IL-1β (*p* < 0.001)], (v) protects mice from *A. baumannii* challenge (survival rate 50%) and (vi) reduces the severity of infection and bacterial burden in lungs of mice (4 log cycles reduction). These results underscore the potential of FilF as a vaccine candidate against rapidly emerging, multidrug resistant *A. baumannii*. FilF efficacy is further being examined by using virulent clinical strains, different animal models and human-friendly adjuvant such as alum. Also, it will be interesting to monitor the immunomodulatory potential of epitopes of FilF and comparison with the complete FilF protein.

## Author contributions

RS: performed all experiments and manuscript writing. NG: established intra-tracheal murine pneumonia model. GS: cytokines estimation and analysis. NC: checked and edited the manuscript. PS: overall guidance, designed the experiments and manuscript writing.

### Conflict of interest statement

The authors declare that the research was conducted in the absence of any commercial or financial relationships that could be construed as a potential conflict of interest.

## Supplementary material

Supplementary Figure S1 **Secondary structure prediction by Phyre2 and GOR IV showing amino acid wise secondary structures of FilF: alpha helix-119 amino acids, extended strand-153 amino acids and random coil-369 amino acids**.

Supplementary Figure S2 **Three-dimensional structure of FilF was predicted by I-TASSER and viewed by SPDBviewer**.

Supplementary Figure S3 **Ramachandran plot obtained from Procheck online tool showing the amino acids residues in plot**.

Supplementary Figure S4 **0.7% agarose gel electrophoresis showing in Lane 1, lambda-HindIII marker; Lane 2, ***filF*** PCR product; Lane 3, purified recombinant pET28a-***filF*** plasmid; Lane 4, pET28a-***filF*** plasmid digested with XhoI; Lane 5, pET28-a plasmid digested with XhoI; Lane 6, Colony PCR for ***filF***, using pET28a-***filF*** as template**.

Supplementary Table S1 **Physico-chemical properties of FilF predicted by ProtParam**.

Supplementary Table S2 **Conservation of FilF among the strains of A. baumannii analyzed by BLASTp**.

Supplementary Table S3 **Genes present in Fil operon**. Fil operon consists of six genes, mostly outer membrane proteins.

Supplementary Table S4 **IEDB prediction of B cell epitopes**.

Supplementary Table S5 **IEDB presiction of MHC I binding epitopes for alleles prevalent in north India**.

Supplementary Table S6 **IEDB prediction of MHC II binding epitopes for alleles prevalent in north India**.
