# Supplementary figures and images for: Immunoprotective Efficacy of Acinetobacter baumannii Outer Membrane Protein, FilF, Predicted In silico as a Potential Vaccine Candidate
Source: Front Microbiol. 2016 Feb 12;7:158. doi: 10.3389/fmicb.2016.00158 (PMC4751259; doi:10.3389/fmicb.2016.00158)

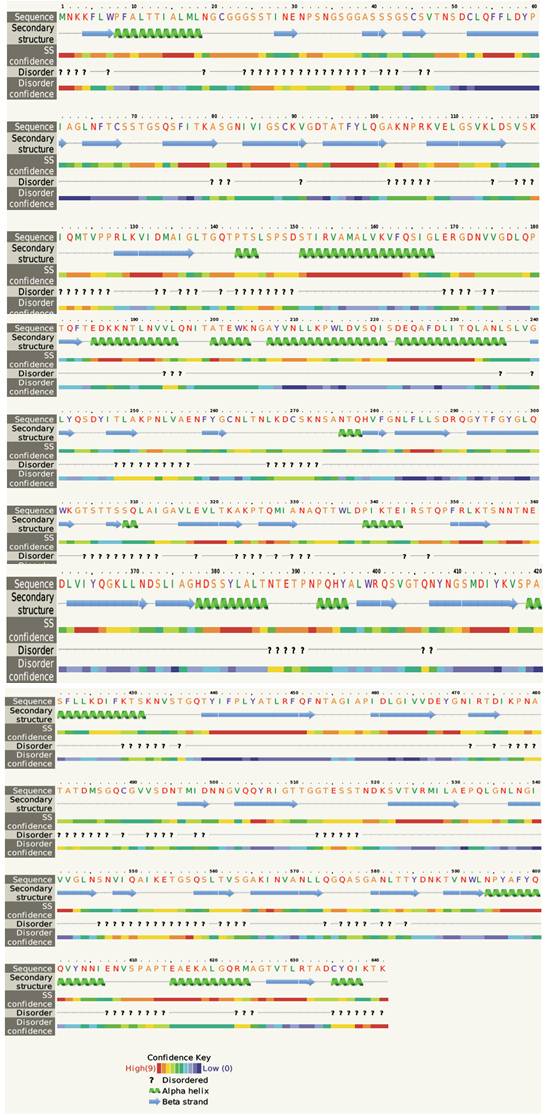

Supplement: Supplementary Figure S1 — Secondary structure prediction by Phyre2 and GOR IV showing amino acid wise secondary structures of FilF: alpha helix-119 amino acids, extended strand-153 amino acids and random coil-369 amino acids. [file Image1.JPEG]

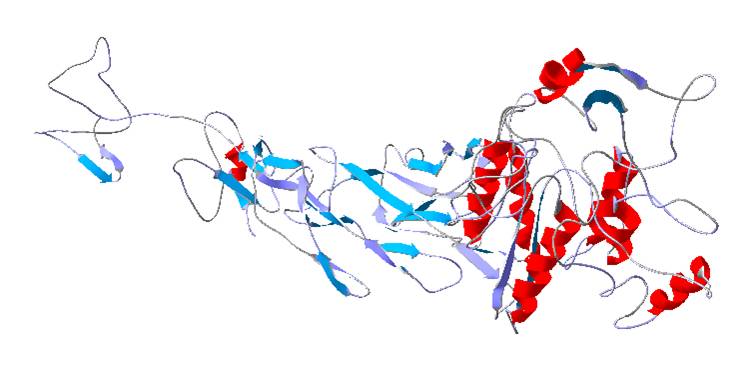

Supplement: Supplementary Figure S2 — Three-dimensional structure of FilF was predicted by I-TASSER and viewed by SPDBviewer. [file Image2.JPEG]

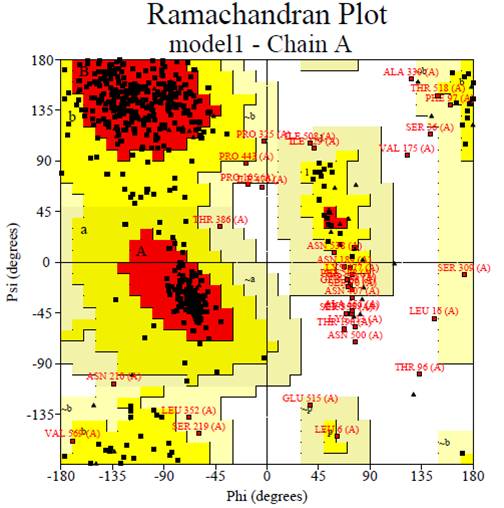

Supplement: Supplementary Figure S3 — Ramachandran plot obtained from Procheck online tool showing the amino acids residues in plot. [file Image3.JPEG]

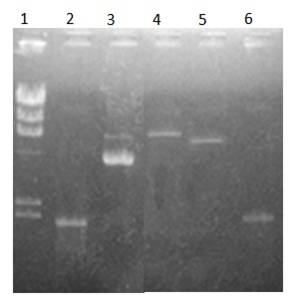

Supplement: Supplementary Figure S4 — 0.7% agarose gel electrophoresis showing in Lane 1, lambda-HindIII marker; Lane 2, filF PCR product; Lane 3, purified recombinant pET28a-filF plasmid; Lane 4, pET28a-filF plasmid digested with XhoI; Lane 5, pET28-a plasmid digested with XhoI; Lane 6, Colony PCR for filF, using pET28a-filF as template. [file Image4.JPEG]
