# Supplementary material for: Immunoprotective Efficacy of Acinetobacter baumannii Outer Membrane Protein, FilF, Predicted In silico as a Potential Vaccine Candidate
Source: Front Microbiol. 2016 Feb 12;7:158. doi: 10.3389/fmicb.2016.00158 (PMC4751259; doi:10.3389/fmicb.2016.00158)
Supplement: Supplementary Table S1 — Physico-chemical properties of FilF predicted by ProtParam. [file Table1.DOCX]

**Suppl. Table S1: Physico-chemical properties of FilF predicted by ProtParam.**

Gene ID: D0C985

Number of amino acids: 641

Molecular weight: 70.51 kDa

Theoretical pI: 5.21

Total number of negatively charged residues (Asp + Glu): 103

Total number of positively charged residues (Arg + Lys): 88

Sub-cellular localization: Outer membrane

Signal peptide: 1-21 amino acids

Adhesion probability: 0.539

Number of trans-membrane helices: 0

Similarity with human, mouse and pig proteome: No

Grand average of hydropathicity (GRAVY) -0.447
