# Supplementary material for: Immunoprotective Efficacy of Acinetobacter baumannii Outer Membrane Protein, FilF, Predicted In silico as a Potential Vaccine Candidate
Source: Front Microbiol. 2016 Feb 12;7:158. doi: 10.3389/fmicb.2016.00158 (PMC4751259; doi:10.3389/fmicb.2016.00158)
Supplement: Supplementary Table S2 — Conservation of FilF among the strains of A. baumannii analyzed by BLASTp. [file Table2.DOCX]

**Suppl. Table S2: Conservation of FilF among the strains of *A. baumannii* analyzed by BLASTp**

| **UniProt ID** | **Protein name** | **Identity** | **UniProt ID** | **Protein name** | **Identity** |
| --- | --- | --- | --- | --- | --- |
| [M2YZJ1](http://www.uniprot.org/uniprot/M2YZJ1) | Pilus assembly protein FilF (Acinetobacter baumannii MSP4-16) | 100.00% | [A0A013MSN3](http://www.uniprot.org/uniprot/A0A013MSN3) | Putative pilus assembly protein FilF (Acinetobacter baumannii 14216) | 99.10% |
| [D0C985](http://www.uniprot.org/uniprot/D0C985) | Uncharacterized protein (Acinetobacter baumannii ATCC 19606 =CIP 70.34) | 100.00% | [A0A062J382](http://www.uniprot.org/uniprot/A0A062J382) | Putative pilus assembly protein FilF (Acinetobacter baumannii 1195185_80) | 99.10% |
| [A0A090BE30](http://www.uniprot.org/uniprot/A0A090BE30) | Protein FilF (Acinetobacter baumannii) | 99.40% | [A0A022JHM5](http://www.uniprot.org/uniprot/A0A022JHM5) | Putative pilus assembly protein FilF (Acinetobacter baumannii 1195185_11) | 99.10% |
| [B0V7P5](http://www.uniprot.org/uniprot/B0V7P5) | Acinetobacter baumannii str. AYE | 99.20% | [A0A011IQX1](http://www.uniprot.org/uniprot/A0A011IQX1) | Putative pilus assembly protein FilF (Acinetobacter baumannii 607805) | 99.10% |
| [A0A0D5YL27](http://www.uniprot.org/uniprot/A0A0D5YL27) | FilF (Acinetobacter baumannii) | 99.20% | [A0A013P0C6](http://www.uniprot.org/uniprot/A0A013P0C6) | Putative pilus assembly protein FilF (Acinetobacter baumannii 214216) | 99.10% |
| [K1FDG6](http://www.uniprot.org/uniprot/K1FDG6) | Uncharacterized protein (Acinetobacter baumannii WC-692) | 99.20% | [A0A013IDX4](http://www.uniprot.org/uniprot/A0A013IDX4) | Putative pilus assembly protein FilF (Acinetobacter baumannii 1413735) | 99.10% |
| [A0A0F4EKM8](http://www.uniprot.org/uniprot/A0A0F4EKM8) | Protein FilF (Acinetobacter baumannii) | 99.20% | [A0A062H5L1](http://www.uniprot.org/uniprot/A0A062H5L1) | Putative pilus assembly protein FilF (Acinetobacter baumannii 1499986) | 99.10% |
| [F5I546](http://www.uniprot.org/uniprot/F5I546) | Uncharacterized protein (Acinetobacter baumannii 6013113) | 99.20% | [A0A011JWC9](http://www.uniprot.org/uniprot/A0A011JWC9) | Putative pilus assembly protein FilF (Acinetobacter baumannii 457946) | 99.10% |
| [F5HUQ3](http://www.uniprot.org/uniprot/F5HUQ3) | Uncharacterized protein (Acinetobacter baumannii 6013150) | 99.20% | [A0A010FDQ9](http://www.uniprot.org/uniprot/A0A010FDQ9) | Putative pilus assembly protein FilF (Acinetobacter baumannii 1096934) | 99.10% |
| [A0A062F7R9](http://www.uniprot.org/uniprot/A0A062F7R9) | Putative pilus assembly protein FilF (Acinetobacter baumannii 754286) | 99.10% | [A0A062EXY2](http://www.uniprot.org/uniprot/A0A062EXY2) | Putative pilus assembly protein FilF (Acinetobacter baumannii 855125) | 99.10% |
| [A0A062DFI1](http://www.uniprot.org/uniprot/A0A062DFI1) | Putative pilus assembly protein FilF (Acinetobacter baumannii 496487) | 99.10% | [A0A062FKH3](http://www.uniprot.org/uniprot/A0A062FKH3) | Putative pilus assembly protein FilF (Acinetobacter baumannii 940793) | 99.10% |
| [A0A013GSK7](http://www.uniprot.org/uniprot/A0A013GSK7) | Putative pilus assembly protein FilF (Acinetobacter baumannii 1170863) | 99.10% | [A0A062RG20](http://www.uniprot.org/uniprot/A0A062RG20) | Putative pilus assembly protein FilF (Acinetobacter baumannii 42057_3) | 99.10% |
| [A0A010T8C8](http://www.uniprot.org/uniprot/A0A010T8C8) | Putative pilus assembly protein FilF (Acinetobacter baumannii 722310) | 99.10% | [A0A009RAM2](http://www.uniprot.org/uniprot/A0A009RAM2) | Putative pilus assembly protein FilF (Acinetobacter baumannii 951631) | 99.10% |
