# Supplementary material for: Immunoprotective Efficacy of Acinetobacter baumannii Outer Membrane Protein, FilF, Predicted In silico as a Potential Vaccine Candidate
Source: Front Microbiol. 2016 Feb 12;7:158. doi: 10.3389/fmicb.2016.00158 (PMC4751259; doi:10.3389/fmicb.2016.00158)
Supplement: Supplementary Table S3 — Genes present in Fil operon. Fil operon consists of six genes, mostly outer membrane proteins. [file Table3.DOCX]

**Suppl. Table S3: Genes present in Fil operon.** Fil operon consists of six genes, mostly outer membrane proteins.


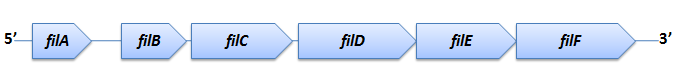


| **Protein** | **Protein ID** | **Locus on contig** | **Length** | **Intergenic length** | **Sub-cellular localization** | **Adhesion probability** | **Trans-membrane helices** | **Similarity with human and mouse proteome** |
| --- | --- | --- | --- | --- | --- | --- | --- | --- |
| FilA | EEX03799.1 | 8733..9602 | 289 aa | 287 bases | Extracellular  (Prob.=0.965) | 0.696 | 0 | no |
| peptidase C39 family protein | EEX03800.1 | 9890..10729 | 279 aa | 29 bases | Unknown  (Prob.=0.2) | 0.277 | 0 | no |
| pilus assembly protein | EEX03801.1 | 10759..11955 | 398 aa | 6 bases | Outer Membrane  (Prob.=0.949) | 0.673 | 0 | no |
| outer membrane transporter family protein | EEX03802.1 | 11962..13632 | 556 aa | 14 bases | Outer Membrane  (Prob.=1) | 0.332 | 0 | no |
| FilE | EEX03803.1 | 13647..14939 | 430 aa | 19 bases | Unknown  (Prob.=0.25) | 0.440 | 0 | no |
| FilF | EEX03804.1 | 14959..16884 | 641 aa |  | Outer Membrane  (Prob.=0.949) | 0.879 | 0 | no |

***filF* coding sequence:**

ATGAATAAAAAATTTTTATGGCCTTTTGCTCTTACCACAATTGCATTGATGTTAAATGGT
TGTGGTGGAGGAAGTTCGACAATTAATGAGAATCCTTCTAATGGATCAGGAGGAGCTAGT
TCTAGTGGAAGCTGTTCTGTAACGAATAGCGATTGTCTACAATTTTTTTTAGATTACCCT
ATTGCTGGTCTTAATTTTACCTGTAGTAGTACTGGAAGCCAAAGTTTTATTACTAAAGCT
TCAGGAAATATTGTCATCGGTTCTTGTAAAGTTGGTGATACAGCGACCTTCTATTTGCAA
GGTGCTAAGAATCCAAGAAAAGTAGAATTAGGTTCAGTTAAGTTAGATTCAGTCTCAAAA
ATTCAAATGACTGTGCCACCTCGTCTAAAAGTCATAGATATGGCAATAGGATTAACAGGG
CAGACTCCAACATCGCTTAGTCCTAGTGACTCTACTATTCGCGTAGCAATGGCATTAGTA
AAAGTTTTCCAAAGCATTGGCTTGGAAAGAGGAGATAATGTAGTTGGTGATTTACAACCA
ACGCAATTTACTGAAGATAAGAAAAATACATTAAATGTTGTATTGCAAAATATCACGGCA
ACGGAATGGAAAAATGGGGCCTATGTCAATTTGTTAAAACCATGGTTAGATGTAAGCCAG
ATTTCAGATGAGCAGGCTTTCGACCTTATAACCCAGTTAGCAAACTTAAGTTTGGTTGGA
TTATATCAATCAGATTATATTACTTTGGCAAAACCTAATTTAGTTGCTGAAAACTTTTAT
GGATGTAATTTAACGAATTTAAAAGATTGCTCAAAAAATAGTGCGAATACCCAACATGTA
TTTGGCAACTTATTTTTGCTGTCTGACCGTCAAGGTTATACATTTGGTTATGGGTTACAA
TGGAAAGGGACCAGCACTACCTCAAGCCAGCTAGCAATTGGAGCTGTGTTGGAGGTATTA
ACAAAAGCTAAGCCAACTCAAATGATTGCCAATGCACAGACTACTTGGCTTGACCCAATA
AAAACAGAAATTAGATCAACTCAGCCGTTCCGTTTGAAAACTTCTAATAATACAAATGAA
GATTTAGTAATTTACCAAGGTAAATTATTAAATGATTCACTTATTGCCGGACATGATAGT
TCGTATTTAGCTTTAACCAATACTGAAACTCCGAACCCACAACATTATGCATTATGGAGA
CAGTCCGTAGGTACCCAAAATTATAATGGCTCTATGGATATTTATAAGGTGAGCCCTGCT
AGTTTCCTCTTAAAAGATATTTTTAAGACTTCTAAAAATGTATCAACAGGCCAAACATAT
ATTTTCCCTTTATATGCAACATTAAGATTCCAGTTCAATACGGCTGGTATTGCACCAATT
GATTTAGGTATTGTGGTTGATGAATATGGGAACATTCGGACTGATATCAAACCTAATGCA
ACGGCGACTGATATGTCTGGGCAATGTGGAGTTGTTTCTGATAATACTATGATCGATAAC
AATGGGGTTCAACAGTATCGTATAGGAACAACTGGAGGAACAGAATCTTCCACAAATGAT
AAATCCGTTACTGTAAGAATGATTCTTGCTGAGCCTCAATTAGGGAATCTGAATGGGATT
GTGGTAGGCCTTAACTCAAATGTCATACAAGCAATTAAAGAAACAGGTAGCCAATCTTTA
ACAGTAAGTGGGGCAAAAATTAACGTTGCCAATCTATTACAAGGTCAGGCAAGTGGTGCA
AATCTTACGACTTATGATAATAAAACAGTTAATTGGTTGAATCCATATGCCTTTTATCAA
CAAGTTTATAACAATATTGAAAATGTCTCACCTGCACCAACTGAAGCTGAAAAAGCACTG
GGACAGCGTATGGCTGGAACAGTAACCCTTAGAACCGCTGATTGTTATCAAATTAAGACA
AAATAA
