# Supplementary material for: Immunoprotective Efficacy of Acinetobacter baumannii Outer Membrane Protein, FilF, Predicted In silico as a Potential Vaccine Candidate
Source: Front Microbiol. 2016 Feb 12;7:158. doi: 10.3389/fmicb.2016.00158 (PMC4751259; doi:10.3389/fmicb.2016.00158)
Supplement: Supplementary Table S4 — IEDB prediction of B cell epitopes. [file Table4.DOCX]

**Suppl. Table S4: IEDB prediction of B cell epitopes**

| **No.** | **Start Position** | **End Position** | **Peptide** | **Peptide Length** |
| --- | --- | --- | --- | --- |
| 1 | 6 | 19 | LWPFALTTIALMLN | 14 |
| 2 | 42 | 71 | SGSCSVTNSDCLQFFLDYPIAGLNFTCSST | 30 |
| 3 | 74 | 79 | QSFITK | 6 |
| 4 | 82 | 92 | GNIVIGSCKVG | 11 |
| 5 | 96 | 101 | TFYLQG | 6 |
| 6 | 107 | 135 | KVELGSVKLDSVSKIQMTVPPRLKVIDMA | 29 |
| 7 | 143 | 150 | PTSLSPSD | 8 |
| 8 | 152 | 167 | TIRVAMALVKVFQSIG | 16 |
| 9 | 172 | 178 | DNVVGDL | 7 |
| 10 | 191 | 198 | LNVVLQNI | 8 |
| 11 | 208 | 221 | YVNLLKPWLDVSQI | 14 |
| 12 | 226 | 259 | AFDLITQLANLSLVGLYQSDYITLAKPNLVAENF | 34 |
| 13 | 277 | 290 | TQHVFGNLFLLSDR | 14 |
| 14 | 308 | 323 | SSQLAIGAVLEVLTKA | 16 |
| 15 | 361 | 368 | DLVIYQGK | 8 |
| 16 | 375 | 386 | IAGHDSSYLALT | 12 |
| 17 | 393 | 401 | PQHYALWRQ | 9 |
| 18 | 414 | 427 | IYKVSPASFLLKDI | 14 |
| 19 | 437 | 451 | GQTYIFPLYATLRFQ | 15 |
| 20 | 457 | 469 | IAPIDLGIVVDEY | 13 |
| 21 | 488 | 494 | QCGVVSD | 7 |
| 22 | 522 | 532 | SVTVRMILAEP | 11 |
| 23 | 538 | 552 | NGIVVGLNSNVIQAI | 15 |
| 24 | 559 | 576 | SLTVSGAKINVANLLQGQ | 18 |
| 25 | 595 | 604 | PYAFYQQVYN | 10 |
| 26 | 608 | 613 | NVSPAP | 6 |
