# Supplementary material for: Immunoprotective Efficacy of Acinetobacter baumannii Outer Membrane Protein, FilF, Predicted In silico as a Potential Vaccine Candidate
Source: Front Microbiol. 2016 Feb 12;7:158. doi: 10.3389/fmicb.2016.00158 (PMC4751259; doi:10.3389/fmicb.2016.00158)
Supplement: Supplementary Table S5 — IEDB presiction of MHC I binding epitopes for alleles prevalent in north India. [file Table5.DOCX]

**Suppl. Table S5: IEDB presiction of MHC I binding epitopes for alleles prevalent in north India**

| **Allele** | **Start** | **End** | **Peptide** | **Percentile rank** |
| --- | --- | --- | --- | --- |
| HLA-A*31:01 | 623 | 631 | RMAGTVTLR | 0.2 |
| HLA-B*58:01 | 293 | 301 | YTFGYGLQW | 0.2 |
| HLA-B*58:01 | 328 | 336 | MIANAQTTW | 0.2 |
| HLA-B*35:03 | 442 | 450 | FPLYATLRF | 0.2 |
| HLA-A*01:01 | 200 | 208 | ATEWKNGAY | 0.25 |
| HLA-A*02:06 | 242 | 250 | YQSDYITLA | 0.25 |
| HLA-A*02:06 | 231 | 239 | TQLANLSLV | 0.3 |
| HLA-A*11:01 | 417 | 425 | VSPASFLLK | 0.3 |
| HLA-B*51:01 | 594 | 602 | NPYAFYQQV | 0.3 |
| HLA-A*01:01 | 357 | 365 | NTNEDLVIY | 0.35 |
| HLA-A*11:01 | 95 | 103 | ATFYLQGAK | 0.35 |
| HLA-C*04:01 | 441 | 449 | IFPLYATLR | 0.35 |
| HLA-A*01:01 | 49 | 57 | NSDCLQFFL | 0.4 |
| HLA-A*01:01 | 287 | 295 | LSDRQGYTF | 0.4 |
| HLA-A*03:01 | 95 | 103 | ATFYLQGAK | 0.4 |
| HLA-B*58:01 | 521 | 529 | KSVTVRMIL | 0.4 |
| HLA-B*40:06 | 53 | 61 | LQFFLDYPI | 0.4 |
| HLA-B*40:06 | 201 | 209 | TEWKNGAYV | 0.4 |
| HLA-A*03:01 | 417 | 425 | VSPASFLLK | 0.45 |
| HLA-B*51:01 | 213 | 221 | KPWLDVSQI | 0.5 |
| HLA-B*51:01 | 311 | 319 | LAIGAVLEV | 0.5 |
| HLA-B*51:01 | 442 | 450 | FPLYATLRF | 0.5 |
| HLA-B*35:03 | 612 | 620 | APTEAEKAL | 0.5 |
| HLA-A*01:01 | 243 | 251 | QSDYITLAK | 0.6 |
| HLA-A*31:01 | 120 | 128 | KIQMTVPPR | 0.6 |
| HLA-A*11:01 | 631 | 639 | RTADCYQIK | 0.65 |
| HLA-A*03:01 | 623 | 631 | RMAGTVTLR | 0.65 |
| HLA-A*01:01 | 588 | 596 | KTVNWLNPY | 0.7 |
| HLA-B*58:01 | 155 | 163 | VAMALVKVF | 0.7 |
| HLA-B*58:01 | 434 | 442 | VSTGQTYIF | 0.7 |
| HLA-B*40:06 | 256 | 264 | AENFYGCNL | 0.7 |
| HLA-A*03:01 | 345 | 353 | RSTQPFRLK | 0.75 |
| HLA-A*02:06 | 130 | 138 | KVIDMAIGL | 0.8 |
| HLA-A*68:01 | 343 | 351 | EIRSTQPFR | 0.8 |
| HLA-B*58:01 | 47 | 55 | VTNSDCLQF | 0.8 |
