# Supplementary material for: Immunoprotective Efficacy of Acinetobacter baumannii Outer Membrane Protein, FilF, Predicted In silico as a Potential Vaccine Candidate
Source: Front Microbiol. 2016 Feb 12;7:158. doi: 10.3389/fmicb.2016.00158 (PMC4751259; doi:10.3389/fmicb.2016.00158)
Supplement: Supplementary Table S6 — IEDB prediction of MHC II binding epitopes for alleles prevalent in north India. [file Table6.DOCX]

**Suppl. Table S6: IEDB prediction of MHC II binding epitopes for alleles prevalent in north India**

| Allele | Start | End | Peptide | Percentile rank |
| --- | --- | --- | --- | --- |
| HLA-DQA1*01:01/DQB1*05:01 | 47 | 61 | VTNSDCLQFFLDYPI | 0.01 |
| HLA-DQA1*01:01/DQB1*05:01 | 48 | 62 | TNSDCLQFFLDYPIA | 0.01 |
| HLA-DQA1*01:01/DQB1*05:01 | 49 | 63 | NSDCLQFFLDYPIAG | 0.01 |
| HLA-DQA1*01:01/DQB1*05:01 | 50 | 64 | SDCLQFFLDYPIAGL | 0.01 |
| HLA-DQA1*01:01/DQB1*05:01 | 51 | 65 | DCLQFFLDYPIAGLN | 0.01 |
| HLA-DRB1*03:01 | 107 | 121 | KVELGSVKLDSVSKI | 0.03 |
| HLA-DRB1*03:01 | 108 | 122 | VELGSVKLDSVSKIQ | 0.03 |
| HLA-DRB1*03:01 | 109 | 123 | ELGSVKLDSVSKIQM | 0.03 |
| HLA-DRB1*03:01 | 110 | 124 | LGSVKLDSVSKIQMT | 0.03 |
| HLA-DRB1*03:01 | 111 | 125 | GSVKLDSVSKIQMTV | 0.03 |
| HLA-DRB3*02:02 | 118 | 132 | VSKIQMTVPPRLKVI | 0.04 |
| HLA-DQA1*01:01/DQB1*05:01 | 52 | 66 | CLQFFLDYPIAGLNF | 0.04 |
| HLA-DRB1*03:01 | 485 | 499 | MSGQCGVVSDNTMID | 0.04 |
| HLA-DRB1*03:01 | 486 | 500 | SGQCGVVSDNTMIDN | 0.04 |
| HLA-DRB1*03:01 | 487 | 501 | GQCGVVSDNTMIDNN | 0.04 |
| HLA-DRB1*03:01 | 488 | 502 | QCGVVSDNTMIDNNG | 0.05 |
| HLA-DRB1*03:01 | 489 | 503 | CGVVSDNTMIDNNGV | 0.05 |
| HLA-DRB3*02:02 | 117 | 131 | SVSKIQMTVPPRLKV | 0.06 |
| HLA-DRB3*02:02 | 119 | 133 | SKIQMTVPPRLKVID | 0.07 |
| HLA-DRB3*02:02 | 411 | 425 | SMDIYKVSPASFLLK | 0.1 |
| HLA-DQA1*01:01/DQB1*05:01 | 46 | 60 | SVTNSDCLQFFLDYP | 0.1 |
| HLA-DRB1*03:01 | 466 | 480 | VDEYGNIRTDIKPNA | 0.11 |
| HLA-DRB1*03:01 | 467 | 481 | DEYGNIRTDIKPNAT | 0.11 |
| HLA-DRB1*03:01 | 468 | 482 | EYGNIRTDIKPNATA | 0.11 |
| HLA-DRB1*03:01 | 469 | 483 | YGNIRTDIKPNATAT | 0.11 |
| HLA-DRB1*03:01 | 470 | 484 | GNIRTDIKPNATATD | 0.11 |
| HLA-DRB3*02:02 | 412 | 426 | MDIYKVSPASFLLKD | 0.15 |
| HLA-DRB3*02:02 | 244 | 258 | SDYITLAKPNLVAEN | 0.18 |
| HLA-DRB1*03:01 | 282 | 296 | GNLFLLSDRQGYTFG | 0.21 |
| HLA-DRB1*03:01 | 281 | 295 | FGNLFLLSDRQGYTF | 0.22 |
| HLA-DRB3*02:02 | 243 | 257 | QSDYITLAKPNLVAE | 0.28 |
| HLA-DRB3*02:02 | 120 | 134 | KIQMTVPPRLKVIDM | 0.3 |
| HLA-DRB1*14:04 | 118 | 132 | VSKIQMTVPPRLKVI | 0.33 |
| HLA-DRB1*03:01 | 283 | 297 | NLFLLSDRQGYTFGY | 0.33 |
